# V2-Specific Antibodies in HIV-1 Vaccine Research and Natural Infection: Controllers or Surrogate Markers

**DOI:** 10.3390/vaccines7030082

**Published:** 2019-08-06

**Authors:** Ralf Duerr, Miroslaw K. Gorny

**Affiliations:** Department of Pathology, New York University School of Medicine, New York, NY 10016, USA

**Keywords:** HIV-1, V2 antibodies, vaccine design, natural infection, non-human primates, correlates of protection, correlates of disease progression, immunogenicity, functional immune response

## Abstract

Most human immunodeficiency virus (HIV) vaccine trials have lacked efficacy and empirical vaccine lead targets are scarce. Thus far, the only independent correlate of reduced risk of HIV-1 acquisition in humans is elevated levels of V2-specific antibodies identified in the modestly protective RV144 vaccine trial. Ten years after RV144, human and non-human primate vaccine studies have reassessed the potential contribution of V2-specific antibodies to vaccine efficacy. In addition, studies of natural HIV-1 infection in humans have provided insight into the development of V1V2-directed antibody responses and their impact on clinical parameters and disease progression. Functionally diverse anti-V2 monoclonal antibodies were isolated and their structurally distinct V2 epitope regions characterized. After RV144, a plethora of research studies were performed using different model systems, immunogens, protocols, and challenge viruses. These diverse studies failed to provide a clear picture regarding the contribution of V2 antibodies to vaccine efficacy. Here, we summarize the biological functions and clinical findings associated with V2-specific antibodies and discuss their impact on HIV vaccine research.

## 1. Introduction

The almost 2 million human immunodeficiency virus (HIV) infections per year worldwide are an incentive to drive progress in the development of a preventive HIV vaccine [1]. The lack of vaccine efficacy in most antibody (Ab)- or T-cell-based vaccine trials and the extreme flexibility and diversity of the HIV envelope protein (Env), the only viral protein facing the immune system before an HIV infection takes place, has made targeted HIV vaccine design a challenging task. Thus far, the RV144 human vaccine trial in Thailand was the only study to report a modest vaccine efficacy in a modified intention-to-treat analysis [2,3]. Despite all the skepticism and controversies about RV144 and the correlates of reduced risk of infection [4,5,6], the proposed lead target, i.e., the hypervariable V1V2 region [2,7], gave direction to the field and provided hope that an Ab-based vaccine is feasible [8,9]. Post RV144, the HIV vaccine field faced a series of unresolved questions that needed to be addressed:

- Can vaccine efficacy and immune correlates be reproduced in other human and non-human primate (NHP) vaccine trials?
- How much did the results depend on the study population, infecting subtypes, and immunogens?
- Were the correlates of reduced risk of infection, and particularly the high levels of V2-specific antibodies (Abs) as the only independent variable, causally linked with protection or were they solely markers for unrelated protective responses?
- How do V2 Abs cooperate with other Abs, other adaptive, and innate immune responses?

Aside from the few human vaccine efficacy trials [9], the majority of HIV vaccine research has been done using NHPs as the best available animal model of HIV/AIDS [10,11,12,13]. NHPs exhibit a high genetic and immunologic homology with humans; e.g., the human and rhesus macaque genomes are ~91% identical (93.5% in the alignable sequence without small indels), and humans and chimpanzees even share ~98.5% of their genomes [14]. Nevertheless, slight differences between humans and NHPs have to be considered when interpreting NHP studies, specifically regarding Ab repertoires and functions [15,16,17,18], as well as challenge viruses, i.e., simian immunodeficiency virus (SIV) versus the humanized version simian-human immunodeficiency virus (SHIV), or HIV.

In addition, studies of natural infection have enabled the investigation of V2 responses that are derived “naturally” after infection with replication-competent primary viruses in the human system. These studies have helped to identify similarities between V2 Ab responses in natural infection and after vaccination. Also, they have enabled to distinguish the requirements for V2 high and V2 low immune responses, to recapitulate the development of functionally potent V2 Abs, to isolate key monoclonal Abs (mAbs), and to identify how V2 Ab responses correlate with other immune responses and clinical outcome [19,20,21,22]. 

Structure/function studies have illuminated structural requirements in defined V2 epitope regions for the binding of at least four different V2 Ab classes [23,24,25,26,27,28]. Env accessibility, inducibility upon immunization or natural infection, and associated antiviral functions are crucial features when assessing biological functions of V2 Abs. The isolation and characterization of V2 mAbs have been critical to demarcate key attributes for different V2 Abs [21,22,29,30,31,32,33,34,35,36,37]. Besides “vaccine-inducible” V2 Abs that are usually non-neutralizing or only weakly neutralize tier 1 viruses, broadly-neutralizing Abs (bnAbs) have been characterized that target glycan-dependent quaternary epitopes in (V1) V2. It has not been possible to induce glycan V2 bnAbs by active vaccination, though germline-targeting immunogen design and successive boosting strategies are currently under investigation to stimulate a directed ontogenetic development of bnAbs [38,39]. Passive vaccination approaches with glycan V2 bnAbs have the promise of complementing combinational treatment protocols [40]. Here, we provide a comprehensive literature review on the biological features of V2-specific Abs. We discuss their potential contribution to vaccine efficacy versus the possibility that vaccine-inducible V2 Abs merely serve as surrogate markers for other immune responses.

## 2. The Multifaceted V1V2 Region and Its Epitope-Specific Abs

**Figure 1:**
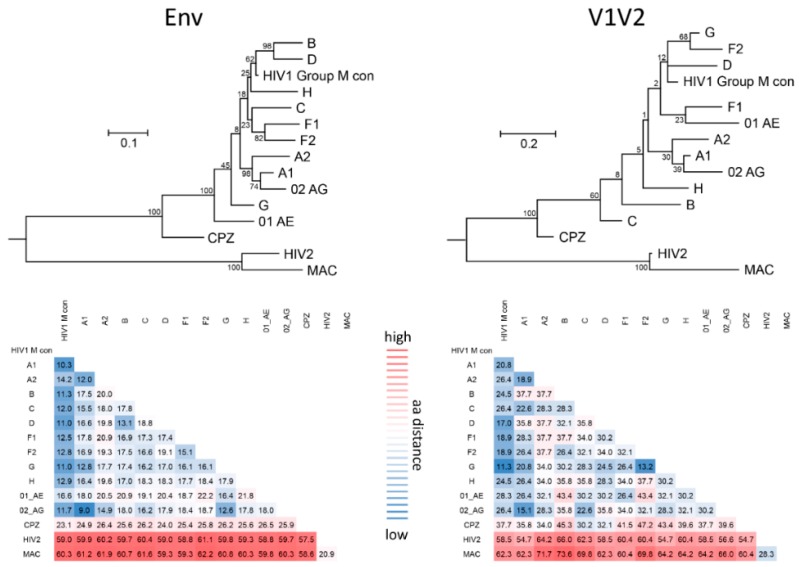
Phylogenetic diversity and aa distance analysis of diverse HIV-1 clades, HIV-2 and SIV. (**Top**): maximum likelihood phylogenetic trees of Env (**left**) and V1V2 (**right**) aa consensus sequences of major HIV-1 clades, HIV-2, and SIV from chimpanzees (cpz) and macaques (mac). An additional consensus sequence summarizing all HIV-1 group M consensus sequences was used (HIV-1 group M con). The consensus sequences were downloaded from the Los Alamos National Library (LANL) Database, if available, or generated from the alignments of functional aa sequences that were used for Figure 2 (Consensus Maker, LANL Database). Maximum likelihood phylogenetic trees were constructed using MEGA v5.2, and the Poisson substitution model with 200 Bootstrap replicates; Bootstrap values are indicated. (**Bottom**): pairwise aa distance analysis between Env (**left**) and V1V2 (**right**) consensus sequences. The heatmap displays aa distances in percent, color-coded according to the scheme in the middle. The distances were calculated in MEGA v5.2 using the Neighbor-joining p-distance model.

**Figure 2:**
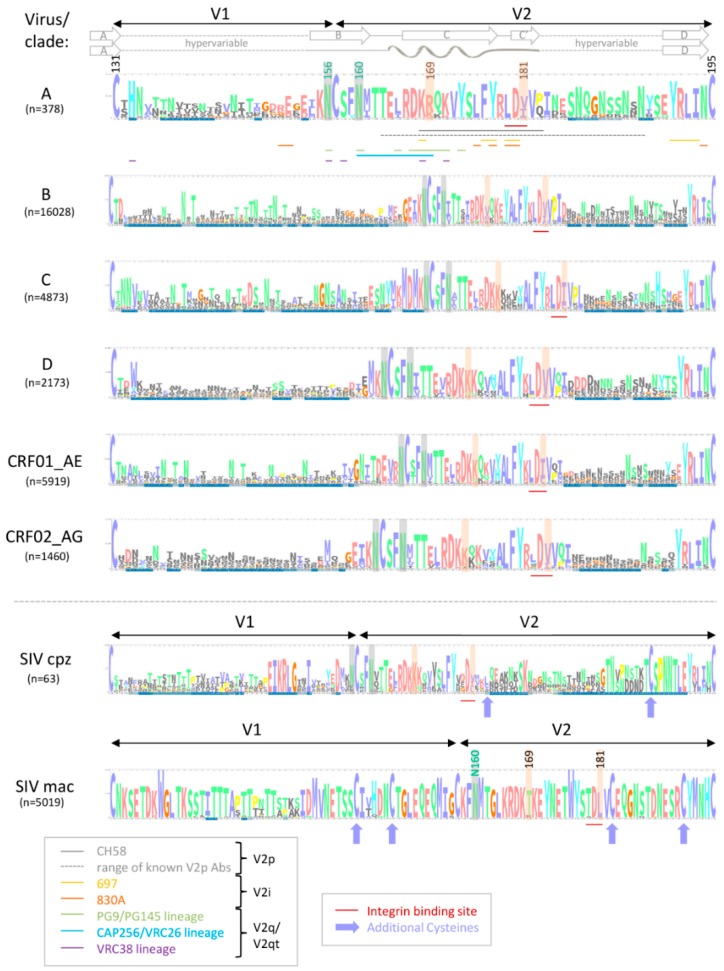
Comparative V1V2 sequence and epitope analysis across major HIV-1 group M clades and SIV. Sequence logo analyses are shown for the six most prevalent HIV-1 group M subtypes and circulating recombinant forms (CRF) in comparison to SIV from chimpanzees (cpz) and macaques (mac). Sequence logos were performed using the Skylign tool (skylign.org) with alignments using all functional aa sequences per virus/clade, downloaded from the LANL Sequence Database. The number of sequences used per logo is shown in parenthesis on the left (n). V1 and V2 loops, their hypervariable regions, and the β-barrel (top; native closed Env; β-strands labeled with A, B, C, C’, and D) versus α-helical conformation (bottom; open Env) are indicated by the schematic at top. The integrin binding site and V1V2 Ab epitopes are indicated with colored lines below the sequence logos according to the box on the lower left. The full set of epitopes is shown for HIV-1 subtype A representatively. Additional cysteines in SIV are highlighted with purple arrows. A dark blue field below the stack of aa indicates occupancy 75% for the respective site. Sites with 5% occupancy were removed from the sequence logos for better clarity. Functionally important aa positions are highlighted: asparagines of the potential N-glycosylation sites N156 and N160 are labeled in green and highlighted with a gray background; sites of immune pressure in RV144 [7], i.e., positions 169 and 181, are labeled and highlighted with an orange background. Epitope and structural regions are indicated according to reference papers and the LANL Immunology Database (Section 2) [22,46,47].

The variable V1V2 region is located at the apex of the HIV-1 Env trimer and contributes to trimer stability, cellular attachment, and viral entry, e.g., via interaction with α4β7 and the CCR5 or CXCR4 co-receptors [24,41]. V1V2 harbors multiple N-glycosylation sites and shields V3 and co-receptor binding sites in the native, closed Env conformation. V1V2 exhibits high structural plasticity and flexibility [42,43] and can transition between different structural states (Section 2.1). There is excessive sequence variation between different viruses and clades (Figure 1). Even within clades, the V1V2 sequence is highly variable, but is interfused with structurally conserved sub-domains, specifically in V2, and these are the main targets for Abs (Figure 2). Plasma V1 Abs are sequence-specific and can be detected almost exclusively against autologous antigens of the infecting virus. In contrast, at least four classes of anti-V2 Abs have been described. They are cross-reactive and detectable in human or NHP plasma samples with heterologous V1V2-fusion proteins and/or V2 peptides [22,35,44,45]. To assess the role of V1V2 and V2-specific Abs in vaccine research, knowledge on the structural V1V2 architecture is essential since the accessibility of the V1V2 epitopes within the native versus open trimer forms affects the antiviral functions of the respective V2 Abs. 

### 2.1. Conformational Plasticity of V1V2 and Structural Requirements for the Binding of Different V2 Ab Classes

**Figure 3:**
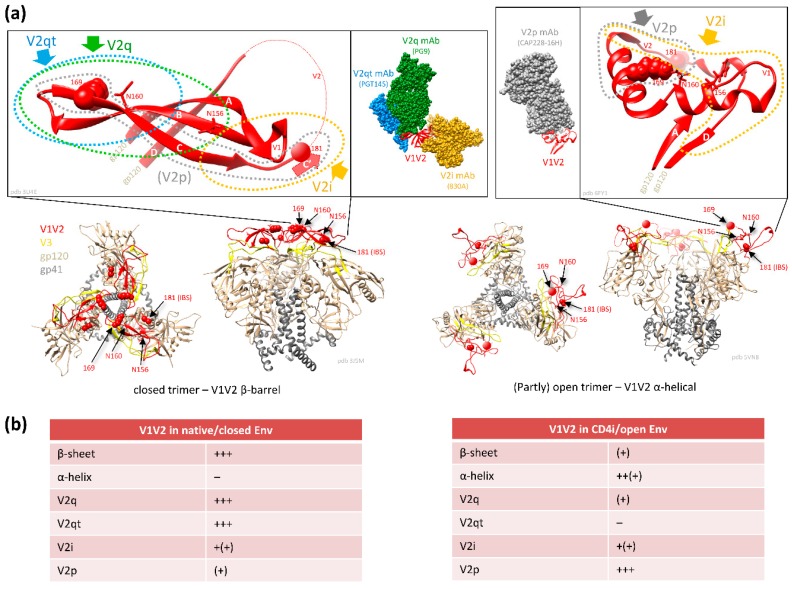
Conformational plasticity of the variable V1V2 region and structural requirements for the binding of different V1V2-specific Abs. (**a**) Trimeric architecture of HIV-1 Env in a closed (pdb 3J5M, Env derived from a PGV04 Fab-stabilized SOSIP co-structure [46]) and an open conformation (pdb 5VN8, Env derived from a b12 Fab-stabilized SOSIP co-structure [52]). Env regions are colored as indicated in the legend; e.g., V1V2 regions are shown in red and V3 regions in yellow. Key V1V2 residues are labeled in one protomer: conserved N-glycosylation sites N156 and N160 are shown in stick representation and sites of immune pressure in RV144 as spheres, i.e., aa 169 and aa 181, the latter being part of the integrin binding site (IBS). Boxed: magnification of V1V2 regions with encircled antigenic V1V2 regions, shown for “closed Env” in preferential β-sheet conformation (left, pdb 3U4E, V1V2 derived from a PG9-stabilized co-structure [53]) and for “open Env” in preferential α-helical conformation (right, pdb 6FY1, V1V2 derived from a CAP228-16H-stabilized co-structure [22]). V1 and V2 loops, gp120 stumps, and the β-sheets A-D are indicated. The binding pattern of different V2 Ab classes is schematically shown with colored ovals and arrows. mAb-bound V1V2 co-structures are displayed in additional boxes. For the V1V2 β-barrel conformation (left) an overlay was generated for PGT145 mAb-bound V1V2 (pdb 5V8L [54]), PG9 mAb-bound V1V2 (pdb 3U4E), and 830 mAb-bound V1V2 (pdb 4YWG [47]) using Matchmaker in Chimera v.1.13.1 (matching restricted to V1V2 regions). Schematics of key regions were manually added if structurally not resolved, i.e., β-strand C’ (arrow), some V1 and V2 loops (red dashed lines), and some aa 169 and 181 residues (spheres). (**b**) Secondary structural content of V1V2 and preferential binding patterns of the different V2 Ab classes to the native/closed versus open Env conformations. Epitope accessibility/binding strength of respective Ab classes is scaled as follows: − absent, + weak, ++ strong, +++ very strong. Parentheses indicate variation within a class. V2 Ab epitopes: V2q: quaternary, V2qt: quaternary and trimer-dependent, V2i: conformational epitope including the IBS, V2p: linear (peptide) epitope. Quantification was done according to reference papers (Section 2.1, Section 2.2 and Section 2.3). SOSIP: soluble, stabilized HIV Env trimers with an additionally introduced disulfide bond (“SOS”) and an isoleucine to proline mutation (“IP”).

V1V2 forms a five-stranded β-barrel structure at the apex of the native closed HIV Env trimer (Figure 3) [46,47]. Upon CD4 activation, the trimer opens, and V1V2 undergoes rotational movements away from the three-fold trimer axis [48]. When the trimer (and V1V2) are released from the constrained closed conformation in native Env, in which V1V2 is mostly found in the β-barrel conformation, V1V2 transitions into a preferential α-helical conformation within the open trimer [22]. The different conformational states can be probed with four kinds of V2 Ab classes (Figure 2 and Figure 3) [49].

V2i Abs (such as 830A) bind a conformational epitope overlapping the integrin binding site and preferentially detect the β-barrel conformation. In contrast, V2p Abs (such as CH58) bind linear V2 peptides corresponding to the C-strand of V1V2 (centered on aa 170–176) and are specific for the α-helix (or helix-coil/coil-helix) conformation. The glycan-dependent, quaternary V2q Abs (such as PG9) preferentially detect the closed native Env conformation with V1V2 in β-barrel conformation, whereof the trimer-dependent Ab subclass V2qt (such as PGT145) is exclusive for closed native trimers (Figure 3) [22,49]. V2q and V2qt Abs are both broadly neutralizing. V2-specific Abs that were suggested as correlates of protection in RV144 belong to the V2i and V2p Ab classes, which are weakly- or non-neutralizing (Section 2.3) [2,50,51].

### 2.2. High Sequence Diversity in V1V2 and Clade-Specific Differences in Antigenicity and Immunogenicity 

One of the major challenges towards the development of an effective HIV vaccine is the enormous viral sequence diversity [55,56]. HIV-1 group M clades reach 20% aa distance in Env, which reaches even 35% in V1V2 (Figure 1). Considering the importance of rhesus macaques as a model system and SIV as frequently applied challenge viruses, it is important to know that SIVmac and HIV-1 group M clades differ up to 60% in Env and 70% in V1V2. A closer look into the V1V2 epitope regions reveals that anchor glycans such as N160 and N156, the lysine-rich region around K169, and the integrin binding site (179–181) are generally present in most viruses/clades, suggesting a conserved biological and possibly antiviral role of V2 Abs. However, the aa frequency distribution at key residues such as aa 169 and aa 181 is highly variable and largely differs by virus/clade (Figure 2). In RV144, viruses matching the vaccine at K169 and having a mismatch at I181 had an estimated vaccine efficacy of 48% and 78%, respectively [7]. K169 has also been linked with the development of strong Ab responses in natural infection and associated with antibody-dependent cellular cytotoxicity (ADCC) or bnAb activities [20,21,44]. Therefore, it is expected that correlates of reduced risk of HIV infection differ considerably when study populations and circulating clades vary. Additionally, the quaternary structure in Env and V1V2 varies considerably between clades. The structural comparison of SOSIP trimers of diverse clades revealed differences in glycan organization, processing, and conservation [57]. Also, the quaternary structural arrangement of the V1V2 regions in two clade C SOSIPs differed substantially to those of clades A and B [58]. As such, the currently ongoing human vaccine trial HPTN702 in South Africa with predominantly clade C infections may significantly differ in outcome from the RV144 trial in Thailand with mostly CRF01_AE infections [3]. 

Compared to other Env regions such as V3, V1V2 is far less immunogenic as evident by the weaker plasma binding levels of V2 Abs in human cohorts of HIV-1 infection and the absence of V2 Ab responses in a fraction of the participants [19,59,60,61]. The detection of V2p Ab responses in HIV-1 infected individuals is less frequent than Ab responses to conformational V2i epitopes [19,20,61,62]. For example, in a Cameroonian cohort, 53–85% of plasma samples were positive against four V1V2 gp70-fusion proteins (preferably detected by V2i Abs), whereas 40–61% were reactive with four V2 peptides (preferably detected by V2p Abs). The screening of sera of HIV-1 infected individuals in clade B cohorts in Europe and the USA yielded even lower V2p reactivities (12% and 21%, respectively) [61,62]. However, V2i and V2p directed immune responses could be readily induced in RV144, presumably supported by using the immunogenic A244 (CRF01_AE) gp120 construct [2,63,64]. Post RV144, diverse V1V2 antigens have been generated that can bind several V2 Ab classes and were able to elicit functional V2i and V2p Ab immune responses upon immunizations in rabbits or rhesus macaques [49,65,66,67,68]. The profiling of linear peptide-binding responses after SIV versus HIV Env immunizations in macaques has shown that HIV-1 induced immune responses focus on V3 with the only exception being the vaccine strain A244, which focuses the response to V2. In contrast, SIVmac239 responses consistently focused on V2 [69]. Furthermore, V1 Ab responses were more frequent in SIV compared to HIV-1 immunizations. The presence of additional disulfide bonds in V1 and V2 of SIVmac is the likely cause for these observations (Figure 2) [70]. Differences in glycan content and composition (complex/high-mannose glycans) and the capacity of Env glycoproteins to stimulate the expression of genes involved in innate or adaptive immune responses in the context of different vectors and adjuvants may have contributed to the observed dissimilarities in immunogenicity between SIV and HIV-1 [38,69,71,72,73,74,75,76]. Of interest, removal of glycans in V1 of SIV gp120 can redirect the humoral immune responses to V3 [75]. Based on these facts, SIV challenge and immunization experiments have to be analyzed with necessary caution considering inherent sequence and immunogenic features of SIV. 

### 2.3. Antiviral Functions Differ According to V2 Ab Class and Epitope Region

Different structural binding modalities of the V2 Ab classes are associated with particular biological and antiviral features. 

#### 2.3.1. Neutralization

Neutralization plays a primary role in vaccine-mediated protection against most viruses, and animal challenge experiments with passive administration of bnAbs indicate that bnAbs can confer protection against HIV if appropriate levels are reached [9,77,78]. However, it has not been possible to induce bnAbs upon vaccination, and the neutralizing Ab responses in RV144 and other human vaccine trials were largely restricted to tier 1 viruses that waned rapidly [79]. 

The majority of vaccine-induced Abs are V2i mAbs that weakly neutralize mostly tier 1 pseudoviruses (Table 1) [31,34,35,65,80] with a neutralizing activity that is similar to other weakly-neutralizing mAbs directed against the CD4-binding site (CD4bs) and the V3 region [81]. In contrast, the few known human V2p Abs are mostly non-neutralizing with the exception of human CH58, CH59, and HGP68, which have modest activity against tier 1 viruses (Table 1) [31,80]. Based on their limited capacity to neutralize a few tier 1 viruses and none of the tier 2 viruses, V2i and V2p Abs are often classified as non-neutralizing Abs [82]. 

#### 2.3.2. V2 Broadly Neutralizing Antibodies

BnAbs occur in a small percentage of HIV-1 infected individuals (10%) after 1–3 years of continuous antigenic stimulation and maturation [83]. A large number of bnAbs have been characterized that mainly target six epitope regions of Env. Key regions are the membrane-proximal external region (MPER) of gp41, the CD4bs, the gp120/gp41 interface, the fusion peptide, the V3 glycan region, and the apical V2 glycan region [84,85,86,87,88]. Depending on the cohort, 10–40% of individuals with bnAb reactivities contain V2 bnAbs [33,89]. V2 bnAbs have been isolated from diverse donors and include PG9 and PG16 [32], PGT141-145 and PGDM1400-1412 [33,90], CH01-04 [91], CAP256-VRC26.01-33 [29,92], BG1 [45], and N90-VRC38.01-11 [93]. V2 bnAbs target a glycan-dependent, quaternary (V2q), and, for some Ab lineages also trimer-dependent (V2qt) epitope at the tip of the trimer (Figure 3). Common features of most V2q/qt bnAbs are their high potency (Table 1) and broad cross-reactivity, combined with a generally low degree of auto-reactivity. In contrast to several other bnAb classes, most V2 bnAbs bind trimeric Env monovalently with only one Ab per trimer at the apex close to the three-fold symmetry axis. They generally possess unusually long CDRH3 loops (≥24 aa) that can penetrate the glycan shield to reach the lysine-rich binding motif in the C-strand of V2 with K169 as a common requirement. These bnAbs are dependent on glycans, specifically N160, for binding, neutralization, and potentially to drive their development [94,95]. V2 bnAbs have an increased propensity for incomplete neutralization (100%) and non-sigmoidal neutralization curves, which has been attributed to glycosylation heterogeneity [96,97]. Stabilization of the V2 loop and/or the closed Env trimer can enhance the presentation of V2 bnAb epitopes [68,98,99,100], yet a certain degree of breathing in the Env trimer is required for efficient binding of V2 bnAbs [54]. Current strategies aim to optimize their usage in passive vaccinations (e.g., Phase 1 clinical trial NCT01937455 for mAb PG9) [40,87] and to recapitulate their sophisticated maturation in vaccine settings using germline-targeting immunogens with guided sequential boosting [39].

#### 2.3.3. Fc-Mediated Effector Functions and Innate Immunity

V2 mAbs can mediate phagocytosis, which correlated with protection in a rhesus macaque study [101], but their activity is moderate and comparable to V2i, V2q/qt, V3, and CD4bs mAbs (Table 1) [65,102,103,104]. ADCC, which is a correlate of immunity in one animal study [105] and a secondary correlate of immunity in the RV144 vaccine trial when corrected for low Env-specific plasma IgA levels [2], can be mediated by V2i, V2p and V2q/qt mAbs (Table 1) [21,31,65,106,107]. Epitope mapping of V2 Abs identified in RV144 indicated that the immune response was largely directed at a linear V2 epitope including K169 [51,108]. Isolation of such K169-directed V2p Abs, after RV144 and in natural human infection, indicated a common usage of the λ light chain complementarity-determining region 2 (CDR2) motif, ED, for binding to K169 [31,109]. The role of V2p mAbs, and to a lesser extent V2i mAbs, in blocking the binding of V2/Env to α4β7 is discussed in Section 5.

While the antiviral functions of vaccine-inducible V2i and V2p Abs are mostly limited in strength, concerted actions of poly-functional immune responses might provide additive functions [110,111,112,113]. V2p Abs isolated from RV144 vaccinees have been shown to synergize with C1 Abs for neutralization, infectious virus capture, and ADCC, achieving ADCC activities at CH58 concentrations similar to the ones detected in the plasma of RV144 vaccinees [114]. The importance of innate immune responses seems to be more and more evident, and are supported by the facts that (i) vectors and adjuvants with improved capabilities to activate innate immune responses affect vaccine efficacy, (ii) macaque challenge experiments suggest innate immune responses as correlates of protection, and (iii) V1V2-specific complement activating serum immunoglobulin G (IgG) significantly, though weakly correlated with inverse infection risk in RV144 [110,115].

## 3. Human Vaccine Efficacy Trials and the Role of V2 Abs

### 3.1. RV144 Reassessed in the Context of Other Human Vaccine Trials

The six completed human HIV-1 efficacy (Phase III) and proof of concept (Phase IIb) vaccine trials indicated that the development of an Ab-based vaccine might be more likely than a T-cell-based vaccine (Table 2). Both T-cell-focused trials, i.e., STEP and Phambili, were stopped due to an increased risk of infection in vaccinated participants, particularly observed in the STEP trial [116,117]. Among the four other vaccine trials, which included the envelope region as immunogen/vaccine target, the vector prime/gp120 protein boost regimen in RV144 proved superior to the gp120 protein-based trials VAX003 and VAX004 and the DNA/vector-based trial HPTN 505 [3,118,119,120]. RV144 was the only study that achieved moderate vaccine efficacy of 31.2% after 3.5 years follow-up in a modified intention-to-treat analysis [3,5]. Elevated levels of V2 Abs against V1V2 fusion proteins or V2 peptides were identified as the only independent variable that correlated with reduced risk of infection and sieve analysis identified signatures of selection pressure in the V2 region of breakthrough viruses [2,7,50,51]. Low plasma IgA levels in combination with ADCC responses were suggested among other secondary parameters (Section 3.2) as possible effector mechanisms in conferring protection [2,31,51,121], yet a complete mechanistic explanation remains elusive. V2 Ab responses in HPTN 505 and VAX004 were low in magnitude and infrequent. In contrast, both VAX003 and RV144 had strong and frequent V2 Ab immune responses in the majority of vaccinees, which, surprisingly, were generally stronger in VAX003 compared with RV144 [2,63,118]. Detailed serological analyses revealed that more than four gp120 protein boosts in VAX003 drove functionally more anergic IgG2 and IgG4 responses, whereas, in RV144, IgG1 and IgG3 responses dominated and were associated with poly-functional humoral effector functions [122,123,124,125]. IgG3 responses were considered particularly critical based on the pinpointed efficacy of fractionated V2 IgG3 [125] and the efficient elicitation of IgG3-mediated Fc-effector functions and complement-activation in HIV and other viral infections [123,126,127]. Of interest, Ab immune responses waned over time in both studies. In VAX003, IgG responses peaked after boosts 3 and 4 and declined after boosts 5–7. In RV144, IgG responses peaked after the second and final protein boost. Subsequently, the V2 Ab response rate against cyclic V2 peptides declined from 97% at two weeks post-immunization to 19% at 28 weeks post last vaccination with a 10-fold drop in binding titers [63,122,124]. It remains open whether this drop in response rate contributed causally to the decline in vaccine efficacy from ~60% at one year compared to ~30% at 3.5 years after study initiation. Notably, additional late boosts that were administered to HIV-uninfected RV144 vaccinees yielded only a short-lived increase of Ab titers to gp120 and V1V2 after the first additional boost that waned rapidly after the second immunization [128]. The high-risk study population of injecting drug users (IDU) in VAX003 has to be considered as a possible reason for the elevated risk of infection compared to RV144. In contrast, RV144 was conducted in the low-risk heterosexual population of Thailand, and 89% of infections in RV144 occurred with CRF01_AE strains, which are supposed to inherit a naturally more open Env configuration [129]. Moreover, boosting in RV144 was performed with a slightly modified gp120 protein (N-terminal 11 aa deletion, replaced with a gD protein-derived tag) of the highly immunogenic A244 strain, which led to the exposure of V2i and V2p epitopes for efficient elicitation of both Ab classes [130]. The qualitatively different V2 Ab immune response in RV144 compared with VAX003 might have been due to the usage of a gp160 DNA prime in a canarypox vector, the more immunogenic CRF01_AE A244 and TH023 antigens, and a better sequence match between the immunogens and the regionally prevalent challenge viruses in RV144 [108,130,131]. 

### 3.2. Alternative Correlates of RV144 Vaccine Efficacy with or without the Contribution of V2

One of the most puzzling findings of RV144 has been the identification that high Env-specific plasma IgA levels were associated with an enhanced risk of infection. Plasma IgA Abs were shown to interfere with functional IgG Abs directed against key epitope regions in RV144, such as C1 [2,121,125,132]. The importance of functional Fcγ-receptors in Ab-mediated protection of SHIV infection is well known [133]; however, ADCC and antibody-dependent cellular phagocytosis (ADCP) have only rarely been identified as an immune correlate of protection in recent macaque challenge experiments (Table 3). Notably, Fcγ-phenotyping revealed that single-nucleotide polymorphisms (SNP) in the FcγR2C gene (CT and TT) conferred 91% vaccine efficacy against K169 viruses in RV144 compared to a 15% vaccine efficacy in individuals with a different SNP (CC) [134]. Of interest, these SNPs are more prevalent in Africa compared to Thailand, which may affect vaccine efficacy in this arm of protection in the upcoming vaccine trials in South Africa [135]. In addition to host factors and Ab-mediated effector functions, functional CD4^+^ T cell responses have been associated with inferred risk. Specifically, Env-specific poly-functional CD4^+^ effector memory T cells with the capacity to produce multiple cytokines, such as CD40L, IL-2, IL-4, IFN-γ and TNF-α were identified as a strong beneficial factor. Dominant IFN-γ responses were identified in CD4^+^ T cells stimulated with CRF01_AE V2 peptides [2,136]. These data suggest that a concerted interplay of vaccine-induced humoral, cellular, cytokine, and host factors was responsible for the partial vaccine efficacy in RV144.

### 3.3. Translation of RV144 Findings into the Development of Future Human Vaccine Trials 

More mechanistic insights into correlates of protection are expected to come from the running vaccine trials in South Africa, i.e., HIV Vaccine Trials Network (HVTN) 702 and HVTN 705/HPX2008 (Table 2). HVTN 705/HPX2008 applies mosaic HIV immunogens with the goal to induce immune responses capable of recognizing diverse globally circulating viral variants. The preparatory Phase I/IIa APPROACH study yielded robust humoral and cellular immune responses, comparable to ones in a matched rhesus macaque challenge study, which achieved 67% protection from SHIV acquisition [137]. HVTN 702 is a further development of RV144 in which the immunogens were adapted to the circulating clade C strains in South Africa, and the adjuvant was changed to MF59 to increase immunogenicity. The vaccine trial will help to clarify whether the increased immunogenicity of MF59 will prove beneficial in humans or whether the vaccine-elicited innate and adaptive immune responses will be skewed towards less protective phenotypes as observed in a recent SIV vaccination study in macaques (Section 4.2) [73]. The preparatory HVTN 100 Phase I/IIa study (same protocol as HVTN 702, applied to a smaller number of participants) achieved strong and cross-reactive IgG binding responses, which qualified the regimen for efficacy testing in the successive Phase IIb/III trial. V1V2 binding responses were lower in magnitude and positivity compared to RV144 and also to HVTN 097, a Phase I study designed to evaluate the RV144 regimen in South Africans [138,139,140]. Fc-mediated effector functions, e.g., ADCP on V1V2 coated beads were also lower in magnitude, which highlighted the importance of the insert sequence for vaccine-elicited Ab immune responses. 

In view of the upcoming vaccine trials, the viral diversity in a study population may have a significant impact on vaccine efficacy. As such, the CRF01_AE diversity in Thailand was less pronounced than the clade C diversity in South Africa. Interestingly, the similarity between the clade C protein boost sequences (1086 and TV-1) in HVTN 100 and HVTN 702 and the circulating strains in South Africa is ~8% more distant from each other than the respective RV144 immunogens and prevalent strains in Thailand [141]. On the contrary, South African clade C viruses have a higher frequency of α4β7-sensitive sequences compared to other clades/geographic regions but the consequences with regards to vaccine efficacy remain to be determined (Section 5) [142]. Regarding the regionally and globally evolving HIV-1 diversity, immunogen sequence adaptations may need to consider the regional predominance of circulating strains as well as the chronological phasing of vaccine trials [9,56].

## 4. The Impact of V1V2-Specific Abs in NHP Experiments

### 4.1. Passive Immunization Experiments

The passive transfer of Abs to animals followed by virus challenge(s) is a well-established method to determine their antiviral functions. Such experiments have demonstrated that Abs against HIV-1 Env are critical for the protection against HIV-1 [143,144,145], SIV [146], and SHIV [147,148]. 

To test the protective function of weakly-/non-neutralizing anti-V2 mAbs, a single V2i mAb was administered to rhesus macaques with subsequent mucosal SHIV_BaL.P4_ challenge [149]. The tested mAb, 830A, weakly neutralized 4 of 41 analyzed tier 1 pseudoviruses with IC_50_ ranging from 0.4 to 36 µg/mL [35]. Passive transfer of 830A mAb resulted in reduced plasma viral load and virus levels in peripheral blood mononuclear cells (PBMCs) and decreased viral DNA in lymphoid tissues, but did not significantly reduce the number of infected animals (5/18 protected or tightly controlled) compared to the dengue virus control mAb [149]. 

These results are similar to three passive immunization experiments in rhesus macaques that tested the protective functions of non-neutralizing mAbs. Whereas a combination of neutralizing mAbs, 2G12, 2F5 and 4E10, specific to gp120 high mannose carbohydrates and gp41, prevented SHIV_SF162P3_ vaginal transmission, the non-neutralizing gp41 mAbs 246-D and 4B3 reduced plasma viral load but had no impact on SHIV acquisition [150]. Another study revealed that neutralizing anti-CD4bs mAb b12 provided sterilizing immunity against SHIV_SF162P4_ challenge in seven of seven macaques, while the non-neutralizing CD4bs mAb b6 did not protect any of the five studied animals. Non-neutralizing anti-gp41 mAb F240 achieved protection in two of five passively immunized animals, thus yielding a limited but overall non-significant reduction of infected animals [151]. The third study demonstrated that two non-neutralizing mAbs, 7B2 (anti-gp41) and A32 (anti-C1), administered passively to rhesus macaques did not protect against SHIV_BaL_ virus infection, contrary to control mAb CH22 (anti-V3), which prevented infection in 4 of 6 animals [152]. These four passive immunization experiments indicate that non-neutralizing HIV-1 mAbs, including anti-V2 mAbs, do not achieve significant protection against virus challenge; however, they can reduce viral load.

In contrast to non-neutralizing Abs, many bnAbs effectively protected rhesus macaques from SHIV infection in a dose-dependent manner, including cases of sterilizing immunity with a single intravenous dose. High efficacies have been reported for engineered bi- and tri-specific bnAb constructs and for bnAbs targeting the CD4bs, the MPER region, the outer domain glycans, the V3 glycan, and the V2 apex region [78,147,148,153,154,155,156,157,158,159,160,161,162]. Among V2 bnAbs, PG9 (V2q), PGDM1400 and CAP256-VRC26.25-LS (both V2qt) have been tested for their protective capacity [159,162]. PG9 achieved only partial protection against the tier 1 virus SHIV_BaL.P4_ at 5 mg/kg, despite a mean neutralization IC50 of 0.06 mg/mL against the same virus, whereas VRC01 and 10E8 were fully protective at the same Ab concentration [159]. In contrast, complete protection against a single high dose challenge with tier 2 SHIV_325c_ was achieved at low serum Ab concentrations 0.75 mg/mL for V2qt mAb CAP256-VRC26.25-LS [162].

Despite the promising results of many passive bnAb SHIV protection studies and a report on vaccine-induced protection from homologous SHIV challenges, which depended on serum nAb titers [163], it is not yet clear whether nAb titers define protection in the heterologous setting of active immunization. Additionally, infections have been observed despite potent neutralization responses of serum antibodies against breakthrough viruses [164]. Besides neutralization per se, Fc-mediated functions play an essential role in Ab protection against HIV infection [133]. There is significant overlap between the capacities of Abs to mediate ADCC against HIV-infected cells and to neutralize viral infection [165,166,167,168]. However, Fc-mediated functions may be partially redundant for very potent bnAbs [169], and ADCC can be uncoupled from neutralization for Abs with low-affinity binding to Env [170]. Though bnAbs have entered the stage of clinical testing, it is not yet known to what extent they can confer protection from HIV-1 infection in humans. Ongoing passive vaccination trials, which are most advanced for CD4bs bnAbs VRC01 (HVTN 703/HPTN 081 and HVTN 704/HPTN 085) and 3BNC117 will provide more information about the protective potential of bnAbs in humans [87].

### 4.2. Vaccine Protection Experiments

Protection experiments in rhesus macaques are important pre-clinical studies to test the efficacy of candidate HIV vaccines. Experiments using SHIV as a challenging virus are the closest model for evaluating the potential of candidate HIV vaccines to generate protective Ab responses in NHPs. The SIV model is more distant from the human system but provides useful data for immune correlate analyses. Some of these NHP experiments analyzed the relationship between the level or titer of vaccine-induced anti-V2 Abs and the outcome of viral infection. 

Only one of six SHIV studies identified a correlation between higher anti-V2 Ab titers and viral control. V2 Ab binding significantly correlated with viral control in the arm where animals were immunized with the replicating adenovirus (SAd7) vector (40% protection), but not in the arm using non-replicating Ad4 (30% protection) [171]. Several other HIV Env/SHIV studies also achieved partial protection of the immunized rhesus macaques against SHIV challenge, which was in the range between 18% and 67%. However, a correlation between anti-V2 Abs and reduced virus acquisition was not observed [101,105,137,172]. The immune correlates differed in each study and included titers of Abs to homologous and heterologous Env proteins (gp120 and gp140), neutralization of HIV_SF162_, Env-specific CD4^+^ T cell responses, ADCC, phagocytosis, and MIP-1β in NK cells (Table 3). Though not protective against SHIV_SF162P3_ challenges, a recent study employed sublingual/buccal and intradermal/subcutaneous immunizations with MVA-HIV DNA and trimeric cycP-gp120 boosts, which significantly delayed the acquisition of infection compared to controls. Strong IgG responses were elicited in serum and mucosal tissues, including broad gp70-V1V2 responses against multiple HIV-1 clades and subdominant V2p responses. Correlates of delayed infection were non-neutralizing Ab effector functions and Env-specific CD4^+^ T-cell responses [173]. Comparably, HIV C.1086 gp140 protein boosts following DNA/MVA vaccination strongly enhanced autologous V1V2-specific immune responses, but they lacked binding to heterologous V1V2 and efficacy against heterologous tier 2 clade C SHIV_1157ipd3N4_ challenge [174].There is one macaque challenge study, which selectively tackled the role of V2i and V2p Abs. Rhesus macaques were co-immunized with V1V2-TH023 DNA, V1V2-A244-2F5K, and V1V2-CaseA2-2F5K fusion proteins, and additionally with cyclic V2-CaseA2 peptides. Two weeks after the last immunization, the animals were challenged with intra-rectal administration of five low doses of SHIV_BaL.P4_ [172]. Four of nine animals were protected from SHIV infection based on undetectable plasma viral load (PVL) and virus DNA in PBMCs and lymph nodes. Plasma Abs did not neutralize SHIV_BaL.P4_ and weakly captured virus without a correlation with PVL. Titers of plasma anti-V2 Abs tested against eight V1V2 fusion proteins and cyclic V2 peptides were high in protected and infected animals without significant differences. In addition to V2 binding, ADCC and phagocytosis were comparable between protected and infected animals and did not correlate with the acquisition of SHIV_BaL.P4_ suggesting that V1V2-directed immunizations can confer partial vaccine efficacy but anti-V2 Abs may not be independently involved in controlling SHIV virus challenge. 

Protection experiments using the SIV Env/SIV challenge model yielded an Ab response that particularly focused on V2. Nine protection experiments were performed in rhesus macaques, which analyzed the titer or level of anti-V2 Abs induced by the SIV Env vaccine towards an SIV challenge. A correlation between titers/levels of anti-V2 Abs and reduced risk of virus infection was common [73,175,176,177,178,179,180,181,182]. Other potential immune correlates were also determined, including anti-Env (gp120, gp140, or cell-bound Env) Abs, Env-specific T cell responses, neutralization, and activation of CD4^+^ monocytes and mucosal NKp44+IL17 cells (Table 3). In one of these studies, a correlation with reduced risk of infection was found for anti-V2 Abs in mucosal secretions, but not in serum [177]. 

Another study used an RV144-like regimen to compare alum, as used in RV144, with the more immunogenic MF59 adjuvant, which is employed in the ongoing HVTN 702 vaccine trial in South Africa. Compared to alum, MF59 induced stronger systemic and mucosal humoral and cellular Env-specific immune responses, both in magnitude and in function (including neutralization, ADCC, and phagocytosis). Surprisingly, only vaccinations with alum reduced the risk of infection (44% vaccine efficacy), which correlated with mucosal anti-V2 Ab levels, whereas vaccinations with MF59 did not achieve significant viral control [73]. Notably, MF59-induced mucosal levels of anti-V2 IgG even correlated with an increased risk of virus acquisition. Between animals of the alum and MF59 arm, differences in Ab glycoforms were detected that are known to impact ADCC and complement activation. These data suggested critical differences in Ab effector functions among antibodies with apparently similar specificities. [73]. In addition to adaptive V2 IgG responses, vaccine efficacy correlated with alum-induced, but not with MF59-induced, Env-specific mucosal innate immune responses. These findings displayed the importance of a fine-balanced interplay between different arms of immunity. The strong dominance of anti-V2 Abs and correlations with viral control in the SIV Env/SIV challenge model supposedly depend on the higher immunogenicity/accessibility of the V2 region in SIV relative to HIV-1. As mentioned in Section 2.2, the SIV V2 region contains a pair of V2 cysteine residues at positions 183 and 191, which plays a role in stabilizing the Env trimer (Figure 2) [183]. Vaccinations of NHPs with SIVmac239 or SIVsmE660 Env antigens induce significantly higher levels of anti-V2 Abs than immunizations with HIV-1 Env including sequences of strains TH023, MN, 1086, ZM651, and A244 [69]. Several studies which observed a correlation between V2 Ab levels/titers and protection from infection suggested that anti-V2 Abs may serve as a surrogate marker for other immune responses [137,175,181]. Studies of natural infection further indicated that the level or titer of anti-V2 plasma Abs could be a marker of an HIV-infected individual’s overall ability to mount Ab responses to Env proteins [19]. Experimental proof with mechanistic explanations for these hypothesis has yet to be provided. Immunizations with SIV appear well-suited to study V1V2-focused immune responses and associated correlates of protection based on the more constrained, and presumably more exposed V1V2 region. These features, however, make SIV significantly different from SHIV/HIV and need to be considered when evaluating experiments with SIV as challenge virus.

## 5. α4β7-Blocking by V2-Specific Abs

V2p, and to a lesser extent V2i Abs, have the unique feature of binding an epitope region, which encompasses the α4β7 integrin binding site. The integrin α4β7 mediates homing of immune cells, including highly HIV-susceptible CD4^+^ T cell subsets to the gastrointestinal tract, which is one of the primary sites of HIV and SIV pathogenesis [184,185,186,187,188,189,190]. HIV Env has been shown to bind to and signal through α4β7 via a conserved tripeptide LDI/V motif in V2 (aa 179–181) (Figure 2 and Figure 3) [191], presumably complemented by secondary binding motifs (e.g., aa 170–173) and unspecific binding mechanisms [192,193,194]. Importantly, α4β7 serves as an attachment factor for HIV and not as an entry receptor, and α4β7 blocking does not neutralize HIV-1 infection [191]. Considering the fact that α4β7-Env binding is not a general feature of all HIV strains [142,195,196,197], α4β7 expression on peripheral CD4^+^ T cells predicts HIV acquisition and disease progression as measured by the increased rates of CD4^+^ T cell decline and higher viral loads [198]. Furthermore, the frequency of α4β7-high memory CD4^+^ T cells correlated with susceptibility to rectal SIV infection [199]. α4β7 increased HIV susceptibility in activated CD4^+^ T cells in an HIV attachment-independent manner [200].

Based on the physiological functions and clinical relevance of the attachment and signaling factor α4β7 on CD4^+^ T cells, a steric blockade of the α4β7-gp120/V2 interaction was suggested as a promising target for drug and Ab therapies, and active vaccinations via induction of blocking V2 Abs [197,201,202]. This led to the hypothesis that anti-V2 Abs in vivo block viral adhesion and subsequent infection of Th17 cells expressing CD4, CCR5, and α4β7 [189,191,203]. This idea received support from a study on anti-α4β7 treatment in a cohort of individuals with inflammatory bowel disease and concomitant HIV-1 infection. The combinational treatment led to a significant attenuation of lymphoid aggregates, which serve as critical sanctuaries for maintaining viral reservoirs [204]. A primatized anti-α4β7 mAb achieved promising results towards virologic control of SIV infection. Blocking α4β7 during acute intravenous SIV infection reduced plasma and gastrointestinal tissue viral loads [205]. When administered before low-dose repeated vaginal challenge with SIV, anti- α4β7 yielded protection in half of the studied animals. Treated animals that eventually became infected had a delay in infection and had decreased damage in the gut-associated lymphoid tissue (GALT) with maintained CD4^+^ T cell numbers [206]. In another study, SIV-infected rhesus macaques that had received antiretroviral therapy (ART) in combination with anti-α4β7 mAb pACT-1 had sustained control of viremia in all animals for more than nine months without therapy [207]. 

By contrast, a humanized version of the same anti-α4β7 mAb (Vedolizumab) neither prevented nor controlled HIV_SF162_ infection of cells in vitro or in humanized mice [208]. More recent studies raised further doubt regarding protective effects mediated by mAbs against α4β7. Administration of pACT1 (mAb agonist of α4β7) or two mAbs against SIV V2 that block α4β7 binding to Env (ITS09 and ITS12) yielded no consistent virologic control in rhesus macaques post-ART [209]. In a small clinical trial with eight HIV infected individuals on ART (NCT0202788175), none experienced sustained suppression of viral load using humanized vedolizumab (anti-α4β7) after ART interruption [210]. These data suggest that the unusual ability of anti-V2 Abs to inhibit α4β7 interaction with gp120 is not responsible for controlling viremia. Synergistic immunomodulatory effects have been observed when used in concert with suboptimal doses of bnAb VRC01, which may be used in future combinational therapies [113]. New generation α4β7-blocking Abs [211] and upcoming vaccine trials in geographic regions with α4β7-reactive viruses [142,193,197,212] will further illuminate the clinical impact of α4β7-inhibition in humans. 

## 6. V1V2-Specific Immune Responses in Natural Infection

Studies of natural infection provide the opportunity to analyze the development of V1V2 immune responses after sustained viral “priming and boosting” in humans [20,21,213,214]. Moreover, it has been the only platform to delineate the maturation of bnAbs [29,44,92,94,95,215,216,217]. Dissecting polyclonal immune responses and viral epitope signatures in natural infection and vaccinees reveals “humoral footprints” that aid vaccine design [19,20,111,218,219,220]. Since human vaccine trials require extensive logistics and financial efforts, animal models are widely used [10,11,12]. However, in comparison to humans, animal models exhibit incongruences in host and viral genomes, immune repertoires, and overall responsiveness. Furthermore, limitations regarding longitudinal follow-up, availability of challenge viruses and immunogens exist [15,16,17]. Thus, studies of natural infection have complemented human and NHP vaccine studies, and there is growing evidence of similarities between immune responses after vaccination and natural infection.

Natural HIV-1 infection is characterized by generally dominant V3 and gp120 Ab responses with subordinate V1V2 responses, caused by the lower immunogenicity of V1V2 compared to V3 [19,63]. Mutational Env analyses suggest that the highly immunogenic V3 loop is more flexible than V1V2. This difference is due to the apical location of V1V2 on the trimer with the three V1V2 protomers associated at the symmetry axis until Env transitions into a CD4-induced open conformation [221]. Nevertheless, V2 Ab responses are detectable in most individuals and are largely based on weakly-/non-neutralizing V2i responses [24,35]. A study of a cohort of chronically HIV-infected subjects showed that the level of anti-V2 Abs, though lower in magnitude and with a broader spread, strongly correlated with the levels of Abs against gp120 and gp41. The titer of anti-gp120 Abs was significantly lower in patients with deficient anti-V2 Abs than in donors with high titer and cross-reactive anti-V2 Abs [19]. The comparison of V2-deficient with V2-reactive immune responses indicated that shorter V2 regions with fewer N-glycosylation sites and higher charge more efficiently trigger a strong V2 Ab response [19]. Another study revealed that in chronic infection, V1V2 length and potential number of N-glycosylation sites increased substantially before declining in late-stage infection. [222]. HIV-1 seems to adjust to host immune pressure by increasing V1V2 length and the addition of shielding glycans. The predominance of shorter V1V2 regions during early and late-stage infection may reflect immature or waning host immunity. These hypotheses were supported by another study showing that longer V1V2 regions with higher numbers of potential N-glycosylation sites protected against HIV-specific neutralizing Abs [223]. V2 Ab responses may also differ with age [20,213] and sex [118,122], and immune responses vary significantly between adults and infants. The immunization of adults and infants with the same Env vaccine yielded a higher frequency of IgG3 V2 Ab responses in infants (43%) compared to adults (12%). Also, vaccine-elicited Env-specific IgA responses were far more frequent in adults than in infants [224]. Maternal V1V2-reactive IgG levels either had no impact or were even associated with increased mother-to-child transmission (MTCT) risk [225]. 

Compared to V2i, V2p Ab responses are less frequent in natural infection (Section 2.2) [19,20,61,62,226]. The main reason is the occluded epitope in the closed native trimer, which carries V1V2 in the five-stranded β-barrel conformation (Figure 3) [226]. Instead, V2p Abs prefer α-helical V1V2 conformations as found in open Env trimers or monomers. Nevertheless, rare V2p-like immune responses have been observed in HIV-1-infected individuals, presumably due to the presence of aberrant forms of Env displayed on the surface of viruses or infected cells, inherently more open conformations of certain strains/clades, better V2 accessibility/responsiveness during acute infection, or the breathing of Env between closed and open states [22,63,129,226,227,228,229]. Consequently, V2p Abs have been isolated both from vaccinees and from naturally-infected individuals [21,22,31]. 

In contrast to V2i and V2p Abs, which can be readily induced in natural infection and vaccinees, broadly neutralizing V2q or V2qt Abs have only been identified in a small percentage of HIV-1 infected individuals (Section 2.3.1 and Section 2.3.2). Neutralization breadth correlates with viral load and CD4^+^ T cell decline, which partly explains why bnAbs have no impact on disease progression in natural infection [230]. The generation of bnAbs requires multiple years of Ab/Env co-evolution [29,44,92,94,95,215,216,217] and can involve superinfection [29]. 

Slower disease progression in natural infection has been associated primarily with host factors, whereas vaccine efficacy is assumed to be associated with humoral responses [9,231]. Recent studies suggest that poly-functional immune responses may be critical both in protection from disease progression and in protection from infection [20,111,218,219]. A recent longitudinal study of an HIV-1 transmission cluster revealed that V2 Ab responses and ADCC (against cells expressing open Env) in the presence of low Env-specific plasma IgA/IgG ratios correlated with slower disease progression [20]. With the caveat of the limited sample size, the identified immune correlates of slower disease progression were similar to the immune correlates of reduced risk in RV144. Notably, V2 binding correlated with the presence of residue K169 in the contemporaneous viruses of the study participants suggesting similar viral hotspot regions for the elicitation of functional V2i and V2p Abs in natural infection and after vaccination [20,21,22,232]. Future studies with higher sample numbers will need to reveal which “humoral and viral fingerprints” in natural HIV-1 infection can be translated into promising vaccine strategies.

## 7. Conclusions

Based on the high plasticity and inter-subtype diversity in the V1V2 region, V2 Ab responses are complex and regionally different. These features may affect the development of an HIV-1 vaccine. V2q/V2qt bnAbs are highly potent in neutralizing a broad variety of HIV-1 strains, but it has not been possible to induce such bnAbs by vaccination. While germline targeting approaches with sequential boosting are still too far from achieving sufficient neutralization breadth and potency, passive vaccination or combinational treatment options with V2 bnAbs may be realistic goals for the next years. In contrast, V2i and V2p Abs that are readily induced by vaccination or natural infection have not yet proven to be equipped/linked with sufficient antiviral activity to confer independent protection from HIV-1 infection. The controversy after RV144 regarding the role of anti-V2 Abs is still sustained. While several NHP studies identified that V2i and/or V2p Abs correlate with protection from infection, most of these studies used SIV as challenge virus, which is more constrained and accessible in V1V2 based on additional disulfide bonds. The majority of SHIV studies did not provide such correlations. Notably, preliminary data of a current NHP study showed 45% protection from SHIV infection after a V1V2-focused DNA prime/protein boost vaccination; however, neither high V2 Ab levels, nor neutralization, ADCC, or ADCP correlated with protection. This and other studies underline the still incomplete picture. It seems V1V2 immunogens can induce partially protective immune responses; however, it is possible that non-specific/innate immune responses are the driving force while V2 Abs serve as a surrogate marker. The ability of V2i and V2p Abs to block α4β7 has attracted much attention. Animal studies and preliminary clinical data confirmed α4β7 signaling as a biologically important pathway; however, V2 Abs did not consistently block the acquisition of HIV-1 infection. 

Based on the currently available data, an independent role for V2i or V2p Abs in conferring vaccine efficacy seems unlikely. Ongoing studies will need to address whether concerted effects of V2 Abs with other Abs and Ab-mediated or innate immune responses influence vaccine impact. Since the antigenic and immunogenic features of V1V2 differ according to clade, vaccine design may need to include regional adaptations. For example, CRF01_AE strains were suggested to inherit a naturally more open Env conformation as compared to most other clades, and thus may be more prone to Ab-mediated clearance. Additionally, CRF02_AG infected individuals were reported to exhibit a similar immunologic pattern of V1V2 responses to those observed in RV144 participants to be associated with slower disease progression. In contrast, V1V2 were shown to be less immunogenic in clades B and C. While SHIV challenge experiments in NHP, including both active vaccinations and passive transfer of V2 Abs, did not provide evidence for a protective role of these Abs, further vaccine studies in humans currently underway will help to determine whether V2-specific Abs can contribute to protection from HIV-1 acquisition and which regional/clade-specific differences should be taken into account for the design of a presumably regional HIV vaccine.

## Figures and Tables

**Table 1:** Ab effector functions according to V1V2 Ab class.

V2 Ab Class/Effector Functions ^1^	V2qt	V2q	V2i	V2p
Neutralization	+++	+++	+(+)	(+)
viral capture	+++	+++	+(+)	+
ADCC	+	+	+	+
ADCP	+	+	+	n.a.
α4β7 inhibition	−	−	(+)	++

^1^ Quantification of effector functions from − absent to + weak, ++ intermediate and +++ strong. Parentheses indicate variation within the Ab class. Quantification according to reference papers as described in Section 2.2; n.a.: no data available.

**Table 2:** Human HIV-1 vaccine efficacy trials and outcome.

#	Trial	Vaccine	Year	Location/Clades/Target Population	Study Number	Ref	Result/Protection	Immune Correlates	V2 Ab Response
I	VAX003	AIDSVAX B/01_AE Gp120	2003	Thailand IDU	2546	[118]	No efficacy	-	Yes, RV144-like, peak Ab responses after 3–4 immunizations, but waning after 5–7 immunizations; relative enhancement of IgG2 and IgG4 responses with limited antiviral functionality
II	VAX004	AIDSVAX B/B Gp120	2003	USA, Canada, Puerto Rico, Netherlands MSM	5417	[119]	No efficacy	-	Yes, lower frequency and titers compared to VAX003 and RV144
III	STEP	MRK-Ad5 B *gag*, *pol*, *nef* (T-cell based)	2007	North America, the Caribbean, South America, Australia MSM	3000	[116]	No efficacy; Immunizations halted; potential for increased risk of HIV infection among Ad5-seropositive, uncircumcised men.	-	No (no Env in vaccine)
IV	Phambili	MRK-Ad5 B *gag*, *pol*, *nef* (T-cell based)	2007	South Africa heterosexual	801 (3000 were planned)	[117]	Immunizations halted after eight months based on STEP trial result. No efficacy at this point	-	No (no Env in vaccine)
V	Thai Prime-Boost/ RV 144	ALVAC-HIV (vCP1521) 01_AE (TH023), AIDSVAX gp120/alum B/01_AE (MN/A244)	2009	Thailand Heterosexual, high risk	16,402	[2,3,7]	Yes, 31.2% efficacy after 3.5 y (60.5% after 1 y)	High titers of V2i and V2p Abs, ADCC in combination with low Env-specific IgA in plasma, viruses with K169 and mismatch at I181	Yes, strong IgG1 and IgG3 responses associated with polyfunctional responses; strong immunogenicity of 01_AE strain A244
VI	HVTN 505	DNA *gag*, *pol*, *nef, env* A/B/C, rAd5 *gag*-*pol* B, *env* A/B/C	2013	USA MSM	2500	[120]	No efficacy; Immunizations halted; no prevention of HIV infection nor reduction of viral load among vaccine recipients who became HIV infected.	-	Low titers and frequency
VII	HVTN702 (The Uhambo Study)	RV144-like, ALVAC-HIV (vCP2438) C (96ZM651), bivalent gp120/MF59 C (TV1 and 1086)	Ongoing, 2016–2021	South Africa adults	5400	https://clinicaltrials.gov/ct2/show/NCT02968849			
VIII	HVTN 705/HPX2008 (The Imbokodo Study)	Ad26 Mosaic (4x) HIV (*gag, pol, env*), gp140/alum protein C	Ongoing, 2017–2022	South Africa women	2600	https://clinicaltrials.gov/ct2/show/NCT03060629			

IDU: Injecting drug user, MSM: Men who have sex with men, y: years.

**Table 3:** Non-human primate vaccine studies with SHIV or SIV challenges.

#	Author	Immunization	Challenge	% Protection	Immune Correlates	V2 Antigens Tested	V2 Abs Correlation
**HIV Env**							
1	Barouch DH Cell, 2013	Ad/MVA (mosaic)	SHIV_SF162P3_	3 chall. 45% 6 chall. 18%	Env Abs Neutral SF162, ADCP	V2 peptides V1V2-gp70	NO
2	Bradley T Nat Comm, 2017	ALVAC/Pentavalent (B and AE clade)	SHIV_1157_ (clade C)	55%	Cell-bound Env Abs, ADCC MIP-1b in NK cells	V2 peptides	NO
3	Barouch DH Lancet 2018	Ad26, gp140 mosaic	SHIV_SF162P3_	67%	Env Abs T-cell response	V1V2-gp70	NO
4	Malherbe DC J. Virol., 2018	Replicating SAd7 Non-replicating Ad4 (1086, clade C)	SHIV_157ipEL_	Sad—40% Ad4—30%	V2 Abs (SAd7)	V1V2 recombinant (1086.C, JRFL, AE244, 14/00/4)	YES—SAd7 NO—Ad4
5	Hessell A/Gorny MK (Keystone abstract 2019)	DNA gp160, AE gp120, clade AE, B	SHIV_BaL.P4_	55%	SHIV capture Abs Neutral. HIV-SF162	V1V2 scaffolds V2 peptides (CaseA2, AE244, 1086, ZM109)	NO
6	Hessell A/Gorny MK (Keystone abstract 2019)	DNA V1V2, AE V1V2 scaffolds, AE, B	SHIV_BaL.P4_	45%	Not determined yet	V1V2 scaffolds V2 peptides (CaseA2, AE244, 1086, ZM109)	NO
**SIV Env**							
7	Barouch DH Nature, 2012	Ad/poxvirus SIVsmE543	SIVmac251 grown in human cells	80%	Env Abs	V2 peptide	YES
8	Roederer M Nature 2014	DNA/Ad5 SIVmac239	SIVmac660	Vaccine efficacy: 69% (mac239)	Env Abs (C3, CD4bs) Neutralization	V1V2mac239	YES
9	Singh S. J. Virol. 2018	DNA, gp120 SIVmac251	SIVsmE660	0%	Neutral. SIVsm660 T cells response	V1V2-gp70 SIVmac251, smE660	YES Mucosal V2 Abs
10	Pegu P. J. Virol. 2013	ALVAC, gp120 SIVmac251	SIVmac251	27% (3 of 11)	Env Abs avidity	V2 peptides SIVmac251	YES
11	Gordon SN. J. Immunol. 2014	HPV, ALVAC, gp120 SIVmac251	SIVmac251	25%	Env-T cells	V1V2 mini protein SIVmac239	YES
12	Gordon SN J. Immunol. 2016	ALVAC, gp120 SIVmac251	SIVmac251	44%	Only V2 Abs	V1V2-gp70 V2 peptides SIVmac251, smE543	YES mucosal V2 Abs-Yes serum V2 Abs-No
13	Kwa S J. Virol. 2015	CD40L DNA MVA SIVmac239	SIVmac251	50%	V2p Abs, gp41 Abs, V1 Abs, gut CD8 T cells	V2 peptides	YES Serum V2p Abs
14	Vaccari M. Nat Med, 2016	ALVAC, gp120 SIVmac251 (alum, MF59)	SIVmac251	44% (alum) 0% (MF59)	Mucosal NKp44+IL17 (alum)	V1V2-gp70 V2 peptides SIVmac239, 251, smE660	YES (Alum, mucosal V2) NO (MF59, mucosal V2 increased risk)
15	Vaccari M Nat Med, 2018	ALVAC, DNA, Ad26 +gp120 SIVmac251, smE660	SIVmac251	52% DNA and ALVAC	Activation CD14 monocytes	V2 peptides SIVmac251, smE543	YES
